# Exploring Levator Palpebrae Superioris Myositis: A Rare Cause of Ptosis

**DOI:** 10.7759/cureus.96722

**Published:** 2025-11-12

**Authors:** Abeer Qasim, FNU Veena, Srikaran Bojja, Sarah Moore, Manjeet Dhallu

**Affiliations:** 1 Internal Medicine, BronxCare Health System, Bronx, USA; 2 Internal Medicine, Rutgers New Jersey Medical School, Rutgers University, Newark, USA; 3 Obstetrics and Gynecology, American University of the Caribbean School of Medicine, Cupecoy, SXM; 4 Neurology, BronxCare Health System, Bronx, USA

**Keywords:** idiopathic orbital inflammatory syndrome, orbital myositis levator palpebrae superioris myositis, ptosis, systemic autoimmune disease, thyroid associated orbitopathy

## Abstract

Ptosis may indicate an underlying serious condition; therefore, careful evaluation and identification of its cause are essential. It can result from various etiologies, presenting as either unilateral or bilateral eyelid drooping. Levator palpebrae superioris (LPS) myositis is a rare inflammatory disorder involving the eyelid-elevating muscle. While orbital myositis commonly affects extraocular muscles, such as the medial rectus, isolated LPS involvement is uncommon.

We present the case of a 47-year-old woman who developed isolated unilateral ptosis and was diagnosed with LPS myositis. The report highlights the typical clinical presentation, diagnostic approach, and management strategies for this unusual condition. Recognition of this rare entity is important, as prompt diagnosis and appropriate therapy can lead to complete recovery and prevent unnecessary interventions. This report emphasizes the importance of considering LPS myositis in patients with isolated ptosis, demonstrates the value of orbital imaging for diagnosis, and illustrates that timely corticosteroid therapy can lead to complete recovery. Recognition of this rare condition can prevent misdiagnosis and unnecessary interventions.

## Introduction

Orbital myositis (OM) is inflammation of the eye muscles, including the levator palpebrae superioris (LPS), and is classified under idiopathic orbital inflammatory syndrome [1]. Typically, OM affects multiple eye muscles, with isolated involvement of the LPS being rare [2]. It predominantly affects females in their 30s to 40s [3]. OM can be classified into a typical acute form, responsive to steroids, and an atypical chronic or recurrent form associated with systemic autoimmune or infectious conditions [4]. Common symptoms include eyelid drooping, diplopia, conjunctival redness, and pain with eye movement [5]. MRI with intravenous contrast is the preferred diagnostic tool [6]. OM may be associated with conditions such as rheumatoid arthritis, systemic lupus erythematosus, sarcoidosis, and anti-neutrophil cytoplasmic antibody (ANCA)-associated vasculitis. Systemic corticosteroids remain the mainstay of treatment [6]; however, more data are needed regarding treatment efficacy across different disease severities and phases. We present the case of a 47-year-old woman diagnosed early by MRI, who showed an excellent response to corticosteroids, supporting the effectiveness of timely intervention.

## Case presentation

A 47-year-old female with a medical history of seasonal allergies presented with left eye ptosis of three days’ duration. The ptosis was initially associated with eye pain, which later resolved. The patient denied any other ocular symptoms, visual disturbances, or trauma. Two weeks prior to presentation, she experienced swelling on the right side of her face, which resolved spontaneously without ocular involvement. She also reported a history of seasonal allergies associated with sinusitis, rhinorrhea, nasal congestion, and watery eyes, and was currently taking mometasone and loratadine daily. She frequently felt fatigued and reported cold intolerance. She denied any past surgical or family history. She also denied any toxic habits, travel history, or drug allergies. 

Upon arrival at the Emergency Department, she had a heart rate of 95 bpm, blood pressure of 126/72 mmHg, and a respiratory rate of 18 breaths per minute. She maintained an oxygen saturation of 98% while breathing room air. On initial physical examination, the cranial nerves (CNs) were preserved, but the patient exhibited left eye ptosis; the rest of the physical examination was unremarkable. Her laboratory findings were significant for anemia; the remaining results are listed in Table 1.

**Table 1:** Workup done and initial labs ANA, Antinuclear Antibody; Anti-DNA Ab, Anti-Double-Stranded DNA Antibody; Anti-mUSK Ab, Anti-Muscle-Specific Kinase Antibody; Anti-CCP, Anti-Cyclic Citrullinated Peptide Antibody; ACE, Angiotensin-Converting Enzyme; TSH, Thyroid-Stimulating Hormone

Test Name	Result	Reference Range
WBC Count	8.9	4.8-10.8 k/μL
RBC Count	4.92	4.00-5.20 MIL/μL
Hemoglobin (HGB)	10.5 (L)	12.0-16.0 g/dL
Hematocrit, Whole Blood	33.8 (L)	42.0-51.0%
Mean Corpuscular Volume (MCV)	68.7 (L)	80.0-96.0 fL
Mean Corpuscular Hemoglobin (MCH)	21.4 (L)	27.0-33.0 pg
Mean Corpuscular Hemoglobin Concentration (MCHC)	31.2 (L)	33.0-36.0 g/dL
Mean Platelet Volume (MPV)	9.1	8.0-12.0 fL
Red Cell Distribution Width (RDW)	19.1 (H)	10.5-14.5%
Iron, Serum	18 (L)	65-175 μg/dL
Ferritin	3.9 (L)	13.0-150.0 ng/mL
Unsaturated Iron Binding Capacity	278	112-346 μg/dL
Platelet Count	399	150-400 k/μL
Lactic Acid Level	1.2	0.5-1.6 mmol/L
Sodium, Serum	140	135-145 mEq/L
Potassium, Serum	4.1	3.5-5.0 mEq/L
Blood Urea Nitrogen, Serum	10.0	6.0-20.0 mg/dL
Creatinine, Serum	0.7	0.5-1.5 mg/dL
Receptor Acetylcholine Receptor Ab	Negative	Negative: < or = 0.30 nmol/L
ANA	Negative	Negative
Anti-DNA Ab	Negative	Negative: < or = 4
Anti mUSK Ab	Negative	Negative: <1:10
Anti-CCP	Negative	Negative: <1:10
Rapid Plasma Reagin (RPR) Screen	Negative	Negative
ACE Enzyme	Negative	Negative
Protein Electrophoresis	Negative	Negative
TSH	3.43 mlU/L	0.40-4.50 mlU/L
C-reactive Protein, Serum	1.74	< or = 5.00 mg/L

Based on the above signs and symptoms, the patient was admitted to the hospital for additional evaluation. Given that she had isolated ptosis, a CT angiogram of the head with and without contrast was performed, which indicated mild intracranial atherosclerosis in the cavernous and clinoid segments of the internal carotid arteries within the anterior intracranial circulation. The posterior intracranial circulation showed no significant abnormalities. 

**Figure 1:**
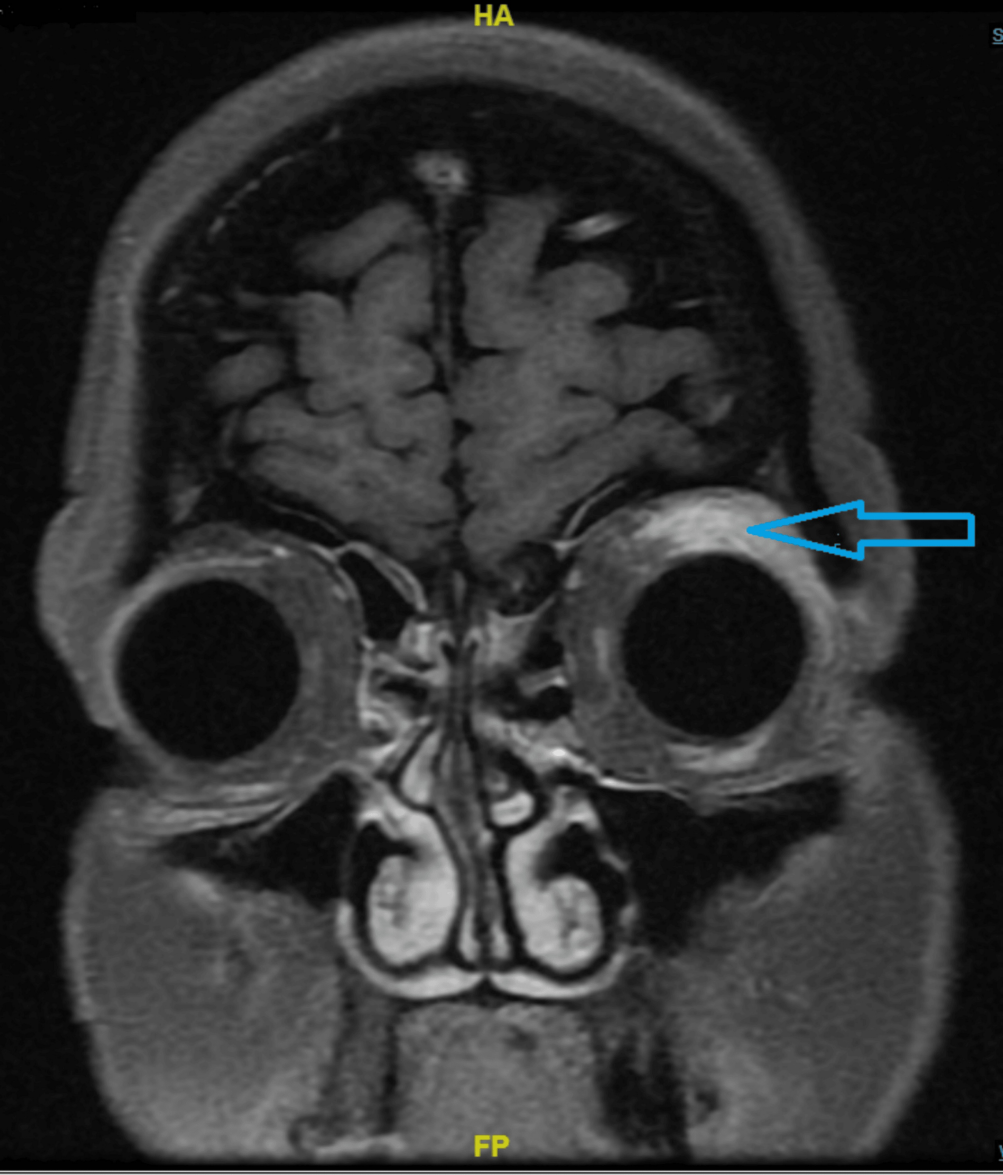
MRI scan MRI revealed T2/STIR (short tau inversion recovery) hyperintensity and focal enhancement of the left levator palpebrae superioris, superior rectus, and adjacent extraconal fat in the left superior temporal quadrant (arrow), consistent with isolated disease.

The neurology and ophthalmology teams were consulted. Slit lamp examination and dilated fundus examination revealed no obvious restriction or limitation in any gaze, and no anisocoria was noted. There was no lid lag or retraction; Cogan's lid twitch was negative. To rule out the possibility of partial third-nerve palsy (pupil-sparing), additional workup was recommended, including MRI of the orbits and MRI of the brain, which indicated an increased signal on T2 and short tau inversion recovery (STIR) sequences within the left LPS, along with focal increased enhancement on postcontrast images. There was no evidence of extension into the retrobulbar structures. In conjunction with the clinical history of left ptosis, these findings were consistent with mild myositis of the left LPS. There was moderate mucosal thickening in the ethmoid air cells, asymmetric mucosal thickening of the left frontal sinus, and mild mucosal thickening in the visualized portions of the sphenoid sinus (Figure 1). 

A trial of Mestinon 60 mg every eight hours was suggested; however, this was not performed since the patient had a negative ice pack test. She was initiated on a prednisone taper regimen (prednisone 40 mg for seven days, followed by 30 mg for seven days, then 20 mg for seven days, and finally 10 mg for seven days), spanning a total duration of 28 days. Repeat examination revealed improving ptosis. During the hospital workup, other possibilities, including myasthenia gravis, PComm (posterior communicating artery) aneurysm, and partial ischemic third CN palsy, were ruled out.

**Figure 2:**
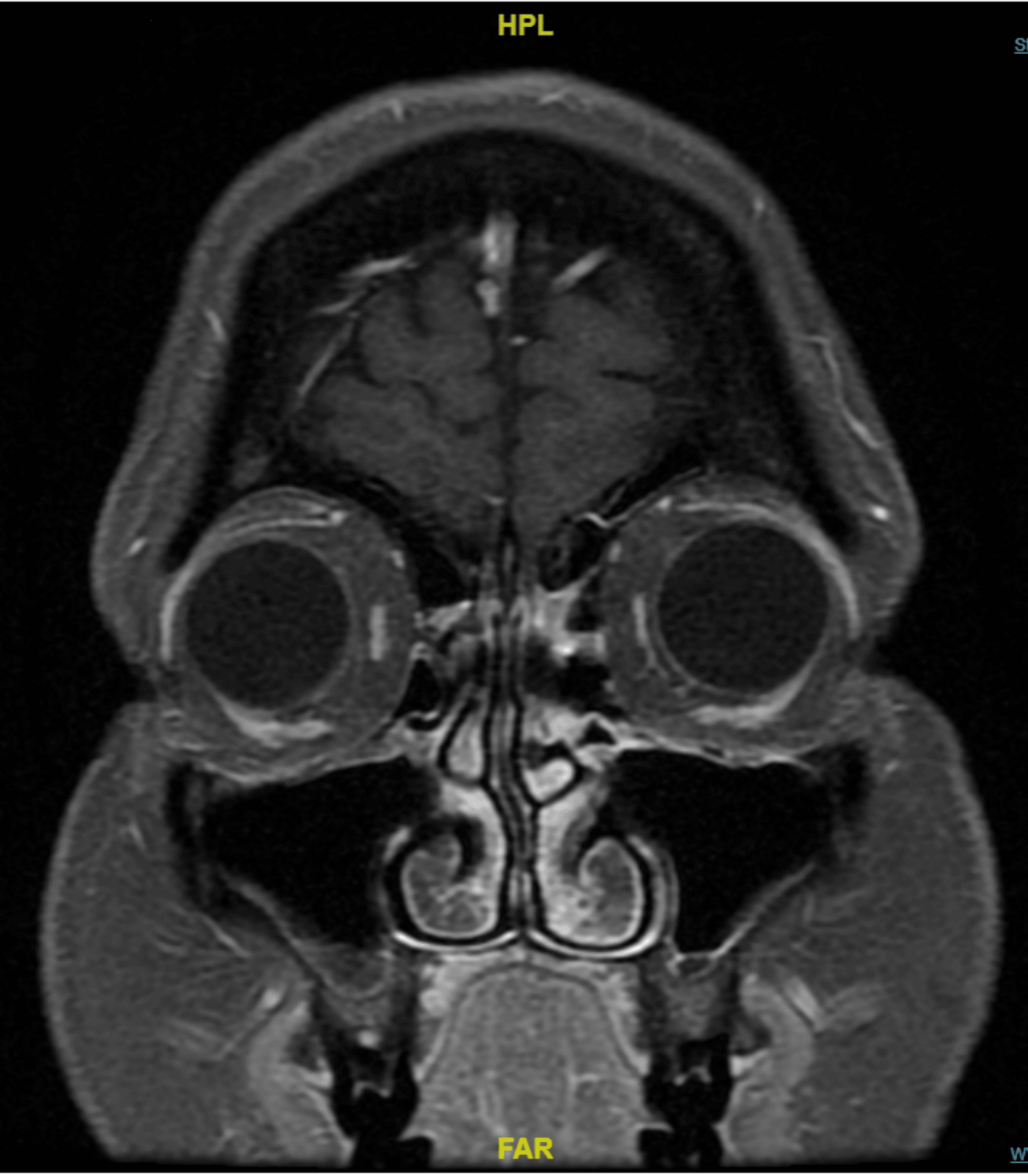
MRI scan The orbits demonstrate resolution of the inflammatory process previously identified in the left superior orbit. The structures now have a symmetric, physiological appearance. The globes are intact. No preseptal abnormalities are found.

ENT evaluation suggested the patient also has chronic sinusitis. Due to concerns for nasocongestion, the patient received a 14-day course of amoxicillin-clavulanate for sinusitis, followed by phenylephrine every eight hours for five days, and pseudoephedrine for five days. Furthermore, they were advised to continue fluticasone propionate on a daily basis as part of their treatment plan. The patient showed significant improvement with the corticosteroids. The patient improved significantly during the follow-up after completion of 10 days of prednisone, with improvement in ptosis. Repeat imaging confirmed improvement in inflammation, as shown in Figure 2.

## Discussion

OM is a rare inflammatory disorder involving inflammation of the extraocular eye muscles. It affects females more than males, in a 3:2 ratio, and typically presents between the ages of 22 and 66 [7]. The incidence of OM varies based on predisposing factors; however, the disease is extremely rare. The most common presenting symptom is unilateral or bilateral painful diplopia. There are two primary forms - one being myositis with additional conjunctival injection only, and the other a more severe myositis involving additional symptoms such as ptosis, chemosis, and proptosis [7]. Most commonly, the medial, lateral, and superior muscles are affected more than the inferior [8]. The differential diagnosis for OM includes thyroid-associated orbitopathy (TAO), ocular myasthenia gravis, Tolosa-Hunt syndrome, oculopharyngeal muscular dystrophy, myotonic dystrophy type 1, congenital cranial dysinnervation disorders, congenital fibrosis of the extraocular muscles (EOM), and late-onset bilateral progressive external ophthalmoplegia [7]. The clinical presentation of TAO is extremely similar to that of OM, as shown in Table 2. Because TAO is one of the leading causes of orbital disease, it is essential to rule it out first [8].

**Table 2:** Comparison of symptoms between orbital myositis and thyroid-associated orbitopathy

Symptoms	Orbital Myositis	Thyroid-Associated Orbitopathy
Ptosis	+	+
Proptosis	+	+
Diplopia	+	+
Unilateral	+	-/+
Bilateral	-/+	+
Orbital Pain	+	+
Chemosis	+	+
Conjunctival Injection	+	+
Systemic Symptoms	-/+	+
Tendon Involvement	+	-

The negative ice pack test in our patient ruled out myasthenia gravis. A negative antinuclear antibody (ANA) result suggested a low likelihood of systemic autoimmune causes, and thyroid function tests (thyroid-stimulating hormone, or TSH) were within normal limits.

The pathogenesis of OM remains poorly understood. However, it is hypothesized that there is an important autoimmune component and that, due to the differences between EOM and other skeletal muscles, the higher blood flow allows inflammatory cells to invade more easily and faster [9]. Ocular myositis is associated with autoimmune diseases including giant cell myocarditis, Crohn’s disease, systemic lupus erythematosus, rheumatoid arthritis, and linear scleroderma [10]. Aside from autoimmune causes of OM, infectious agents have also been attributed to the production of symptoms, such as Lyme disease, group A strep, and herpes zoster virus [10]. Lastly, many cases of OM are idiopathic, with sudden onset and no known preceding symptoms, and a few have been related to episodes of sinusitis [11]. 

Although the diagnosis of OM is essentially a diagnosis of exclusion, patients with similar clinical presentations should undergo laboratory studies including complete blood count (CBC), liver and kidney function tests, thyroid function tests, various antibody tests, erythrocyte sedimentation rate (ESR), C-reactive protein (CRP), rheumatoid factor, and viral markers. Imaging should include chest X-ray, ECG, and echocardiography; ultrasonography of the thyroid and abdomen; thorax CT; and contrast MRI with fat stranding [12]. Typical MRI findings, with increased signal on T2 and STIR sequences, demonstrate thickening of the involved EOM, including the myotendinous insertion. This is another difference between TAO and OM, as OM tends to spare the muscle tendons [9,13]. Rarely, a muscle biopsy is used. Recurrence occurs in about 23% of patients [14], and although most commonly in the same eye, it can present with alternating laterality and different EOM involvement [15]. Risk factors for recurrent disease include male gender, cases with multiple muscle involvement, and lack of response to treatment (specifically corticosteroids) [13]. Other complications related to long-term OM include fibrosis of the affected muscle [7]. On MRI, there is often heightened signal intensity on T2 and STIR sequences, with enlargement of the affected EOM, including involvement of the myotendinous junction. This finding helps differentiate OM from TAO, which typically does not involve the muscle tendons [16]. 

**Figure 3:**
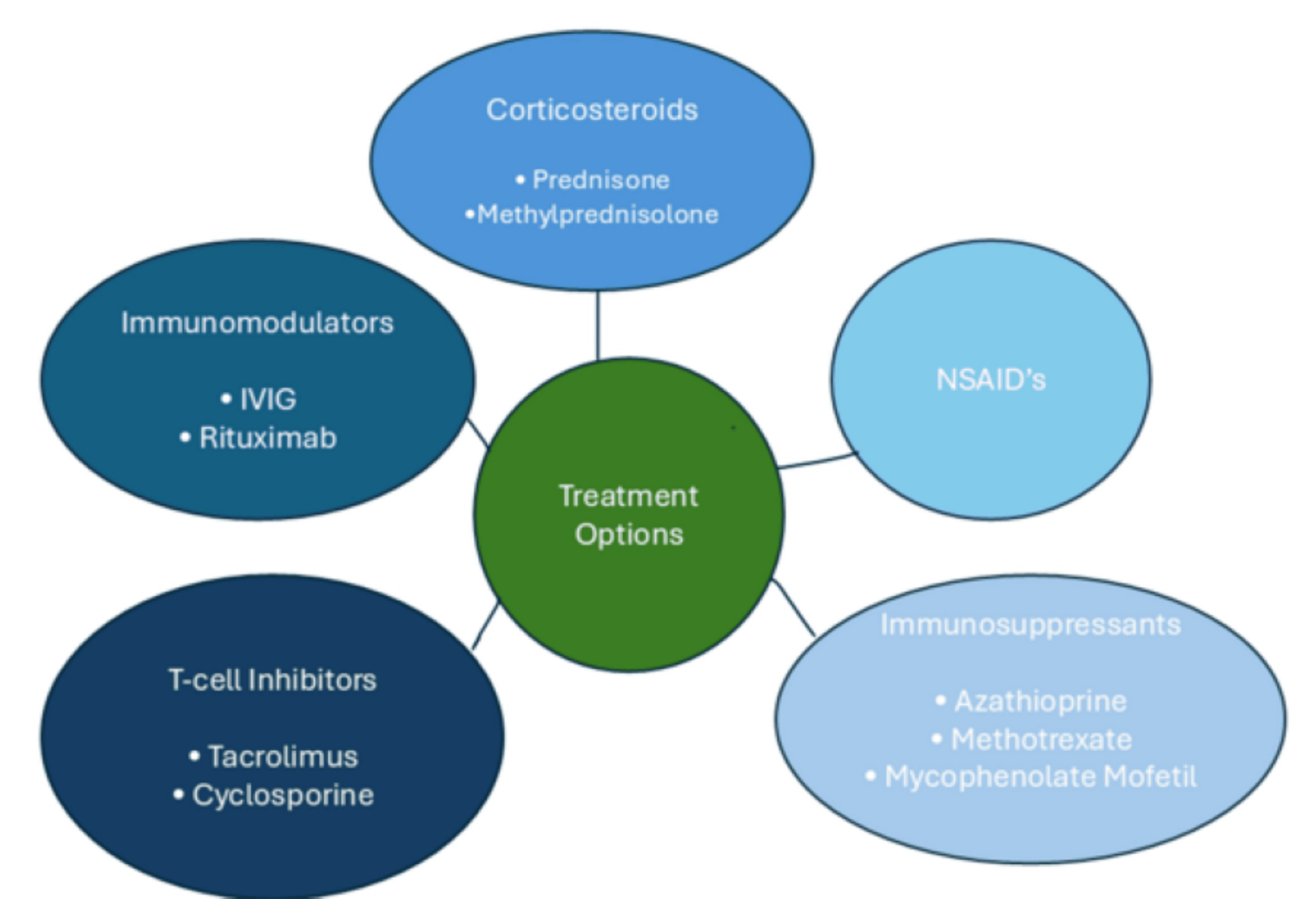
Available treatments that have shown efficacy in cases of orbital myositis

Once a diagnosis has been established, discussing and providing treatment options based on the patient's specific presentation is important. Confirmation of a correct diagnosis of OM can be made with rapid response and improvement of symptoms after beginning steroids. However, due to OM's autoimmune component, recurrence is a likely complication. The first-line therapy is generally a consistent dosage of high-dose corticosteroids for one to two weeks, followed by a taper dosage for 6-12 weeks [7]. Along with steroids, patients who present with other associated autoimmune disorders or previous episodes of recurrence are also started on immunosuppressive medications. Antimetabolites such as azathioprine, methotrexate, mycophenolate mofetil, and T-cell inhibitors such as tacrolimus and cyclosporine have also been used [7]. For patients who do not respond well to corticosteroids or cannot tolerate the adverse effects, some studies have shown methotrexate, IV immunoglobulin, and rituximab as viable options [7,9]. For patients who present with nonspecific, mild disease, non-steroidal anti-inflammatory drugs (NSAIDs) can also be considered as a beginning option for treatment [12]. Because this disease is not entirely understood, many different combinations and trials of treatments are available, like the ones mentioned above and represented in Figure 3. 

The patient described above was presented as a potential textbook presentation of idiopathic OM, with no preceding autoimmune disorder, and had an excellent response to corticosteroid treatment. Her presenting symptoms of ptosis with eye pain, swelling of the LPS extraocular muscle on MRI, and exclusion of thyroid disease based on lab values follow the perfect diagnostic workup. Her associated sinusitis could have played a role in the development of her diagnosis; however, overall, her prognosis is favorable due to the unilateral and singular nature of her eye muscle involvement and her favorable response to treatment. Recurrence has been shown to occur not only months but also years later, with long symptom-free periods, so patients should continue to follow up and report symptoms to catch recurrence early if it does occur [15].

## Conclusions

OM is a rare inflammatory condition of the extraocular muscles that is often diagnosed by excluding other similar disorders, such as TAO or myasthenia gravis. Prompt recognition and treatment - particularly with high-dose corticosteroids - usually lead to rapid symptom improvement and favorable outcomes, as demonstrated in our patient. While most cases resolve without long-term complications, recurrence can occur even years later, especially in patients with multiple muscle involvement or an incomplete response to therapy. Therefore, ongoing follow-up is essential to monitor for recurrence and ensure timely management.
